# Effects of sludge inoculum and organic feedstock on active microbial communities and methane yield during anaerobic digestion

**DOI:** 10.3389/fmicb.2015.01114

**Published:** 2015-10-13

**Authors:** David Wilkins, Subramanya Rao, Xiaoying Lu, Patrick K. H. Lee

**Affiliations:** School of Energy and Environment, City University of Hong KongKowloon Tong, Hong Kong

**Keywords:** anaerobic digestion, biogas, methanogenesis, pyrosequencing

## Abstract

Anaerobic digestion (AD) is a widespread microbial technology used to treat organic waste and recover energy in the form of methane (“biogas”). While most AD systems have been designed to treat a single input, mixtures of digester sludge and solid organic waste are emerging as a means to improve efficiency and methane yield. We examined laboratory anaerobic cultures of AD sludge from two sources amended with food waste, xylose, and xylan at mesophilic temperatures, and with cellulose at meso- and thermophilic temperatures, to determine whether and how the inoculum and substrate affect biogas yield and community composition. All substrate and inoculum combinations yielded methane, with food waste most productive by mass. Pyrosequencing of transcribed bacterial and archaeal 16S rRNA showed that community composition varied across substrates and inocula, with differing ratios of hydrogenotrophic/acetoclastic methanogenic archaea associated with syntrophic partners. While communities did not cluster by either inoculum or substrate, additional sequencing of the bacterial 16S rRNA gene in the source sludge revealed that the bacterial communities were influenced by their inoculum. These results suggest that complete and efficient AD systems could potentially be assembled from different microbial inocula and consist of taxonomically diverse communities that nevertheless perform similar functions.

## Introduction

Microbial anaerobic digestion (AD) of wastewater and sewage allows the recovery of energy in the form of biogas (methane) while simultaneously reducing the concentration of organic substrates and displacing pathogens. This makes it a valuable component of both municipal and industrial wastewater treatment, as on-site energy consumption can be offset by biogas production. While the use of AD to treat wastewater and sewage streams is well-established, it is increasingly considered a viable method for the treatment of solid organic wastes including food waste and the organic components of municipal solid waste (MSW; Zhang et al., 2007; Khalid et al., 2011). These materials would otherwise go to landfill, where microbially mediated aerobic or anaerobic decomposition would release carbon dioxide and methane to the atmosphere, or to incineration with similar consequences. Capturing energy in the form of biogas while simultaneously reducing greenhouse gas emissions thus makes AD an attractive alternative to traditional solid waste management practices (Khalid et al., 2011).

Digestion of sewage sludge amended with food waste can result in higher methane production than from either substrate digested separately (Kim et al., 2003; Sosnowski et al., 2003; Iacovidou et al., 2012), although the improvement is conditional on the mixing ratio and reactor conditions (Heo et al., 2003; Zhang et al., 2008). Lignocellulosic biomass including agricultural by-products (e.g., rice straw, corn stover) and domestic “green waste” (e.g., lawn clippings) are also attractive solid waste amendment candidates due to their high availability, low cost and the environmental impact of alternative disposal methods (Li et al., 2011). However, these materials require pre-treatment with a method such as steam pre-treatment (Chandra et al., 2007), acid hydrolysis or alkali treatment (Galbe and Zacchi, 2007) to separate the cellulose, hemicellulose, and lignin components which are covalently linked and thus recalcitrant to microbial catalysis. These methods typically yield some combination of cellulose, glucose, and xylose/xylan as the major bacterially available components following hydrolysis (Öhgren et al., 2005; Chandra et al., 2007; Galbe and Zacchi, 2007).

Despite the importance and widespread use of AD, the composition of AD microbial communities is poorly understood (Rivière et al., 2009; Narihiro et al., 2015) and major methanogen groups regularly detected in AD reactors remain uncharacterized (Narihiro et al., 2009; Nelson et al., 2011). All known methanogens are of the phylum *Euryarchaeota*, which comprises six established orders (*Methanobacteriales, Methanocellales, Methanococcales, Methanomicrobiales, Methanopyrales*, and *Methanosarcinales*) and one proposed order (*Methanomassiliicoccales*; Borrel et al., 2013). In AD reactors, the hydrogenotrophic *Methanobacteriales* and *Methanomicrobiales* and acetoclastic/hydrogenotrophic *Methanosarcinales* are typically dominant (Nettmann et al., 2008; Rastogi et al., 2008; Zhu et al., 2008; Nelson et al., 2011; Wilkins et al., 2015). The uncultured ArcI/Arc I/WSA2 group is also routinely detected at high abundance in AD communities (Chouari et al., 2005b; Rivière et al., 2009; Nelson et al., 2011; Wilkins et al., 2015). The bacterial component of AD communities is typically dominated by the phyla *Chloroflexi, Proteobacteria, Firmicutes*, and *Bacteroidetes* (Rivière et al., 2009; Nelson et al., 2011). With the exception of *Chloroflexi*, the functional role of which is still being actively investigated, genera detected from the remaining three major phyla are associated with all steps of the AD process excluding methanogenesis (Nelson et al., 2011); functional assessment of the role of bacterial groups in AD therefore benefits from classification to the family level or finer. Notably, a meta-analysis of AD 16S rRNA gene surveys found over 50% of *Bacteroidetes* sequences could not be classified beyond phylum (Nelson et al., 2011). The effects of substrate and inoculum source are also poorly explored. While meta-analyses of AD microbial communities have found that they cluster by substrate (Regueiro et al., 2013; Zhang et al., 2014), it is not clear whether diverse and substrate-specific communities can be enriched from a common source by substrate amendment. A more complete understanding of the microbial communities associated with the digestion of wastewater sludge amended with organic solids is critical to improving the efficiency of this method.

This study aimed to characterize the relative importance and effect of both sludge inoculum and organic waste substrate on the active archaeal and bacterial community composition and methane yields from AD. We sought to determine whether or not the source inoculum continues to have a large effect on community composition and methane production following medium-term enrichment, and if so whether this effect is mediated by the organic waste substrate. Two sludge types (from industrial wastewater and sewage) were incubated with food waste as well as cellulose, xylose, and xylan representing pre-treated lignocellulosic organic matter. Previous studies aimed at characterizing the community composition of AD sludge or wastewater (Chouari et al., 2005a; Rivière et al., 2009; Nelson et al., 2011; Zhang et al., 2014) or of cultures inoculated from AD sources (Wagner et al., 2013; Narihiro et al., 2015) have used clone library or pyrotag sequencing of the 16S rRNA or other marker genes. While this method is able to give an overview of the cells present in the system, it does not differentiate between active (i.e., metabolizing and dividing) and dormant cells. In systems such as AD where sludge and wastewater are recycled and cells may have long residence times, and particularly in closed laboratory cultures, this may exaggerate the importance of inactive populations. In contrast, reverse transcription and sequencing of transcribed small-subunit rRNA provides a more accurate reflection of the metabolically active microbial population. By targeting transcribed rRNA molecules rather than rRNA genes, this study was thus able to reveal the active archaeal and bacterial populations in different digestion scenarios. We also examined the effects of meso- and thermophilic temperatures on the digestion of cellulose.

## Materials and Methods

### Sample Collection

Two sludge samples were obtained for this study. The first was taken from an Upflow Anaerobic Sludge Blanket (UASB) digester treating sugar wastewater from a beverage factory in Guangzhou, China (“GZ”). The second was taken from mesophilic anaerobic digester in the Shek Wu Hui sewage treatment plant in Hong Kong (“SWH”). Operating conditions and physicochemical properties for these digesters have been previously reported (Wilkins et al., 2015). Triplicate 1 L samples were collected simultaneously from the midsection of each digester and mixed thoroughly. The samples were incubated at 35°C and used as inocula within 72 h.

### Batch Culture

A series of batch culture experiments were carried out to identify the major active taxa involved in the digestion of various substrates. Sludge samples (50 mL) from the two digesters were centrifuged at 1,500 × *g* for 2 min, then resuspended in 100 mL of 0.2 M phosphate buffer (pH 7.2) made anaerobic by purging it with ultra-high purity (99.999%) N_2_ gas. Food waste was collected from the university canteen and blended into slurry with a food processor. The volatile solids composition of the food waste was determined by the standard method given in Rice et al. (2012). Cellulose (type 101, highly purified fibers), xylan (from beechwood), and xylose (purity ≥ 99%) were purchased from Sigma-Aldrich (St. Louis, MO, USA). Duplicate batch cultures were set up in 160 mL serum bottles each with 5 g volatile solids/L of food waste or 5 g/L of cellulose, xylan, or xylose as the sole carbon source. Controls with no added substrate were also prepared. Serum bottles were sealed with butyl rubber stoppers and purged with ultra-high purity N_2_ gas for 10 min to ensure completely anaerobic conditions. The serum bottles were incubated at 35°C (i.e., similar to the mesophilic operating temperature of the sampled AD systems) without shaking. To investigate the effect of temperature, additional duplicate cellulose cultures were incubated at 55°C (thermophilic) without shaking. Incubations proceeded for 69–87 days and samples for chemical analysis were collected every 7–10 days.

To determine the total methane production of each culture, headspace gas was collected from each culture and methane concentration measured by gas chromatograph (GC; GC-2010 Plus, Shimadzu, Kyoto, Japan) with a flame ionization detector. The injector and detector temperatures were isothermal at 30 and 200°C, respectively, and the GC was programmed to maintain 35°C for 8 min. Helium (3 mL/min) was used as the carrier gas in a Rt-QS-BOND column (Restek Corporation, Bellefonte, PA, USA). The volume of headspace gas was measured every 3–10 days by syringe at ambient temperature and pressure, and the total volume of methane produced was calculated. Volatile fatty acids (VFAs) concentrations were determined by a high-performance liquid chromatograph fitted with an Aminex HPX-87H column (Bio-Rad, Hercules, CA, USA) and photodiode array detector (Waters, Milford, MA, USA).

### 454 Pyrosequencing and Operational Taxonomic Unit (OTU) Formation

Nucleic acid extraction, reverse transcription, PCR amplification and sequencing were performed as previously described (Lu et al., 2013). Briefly, 1 mL from each duplicate culture was collected and pooled at the midpoint of growth, as determined by linearly increasing methane concentration. Samples were immediately centrifuged at 13,800 × *g* for 6 min at 4°C, and the cell pellet stored at -80°C until RNA extraction (less than 1 week). Total RNA was extracted with the RNeasy Mini Kit (Qiagen, Valencia, CA, USA) following the manufacturer’s protocol, with additional mechanical lysis by vortexing for 10 min with 100 mg of 100 μg-diameter zirconia/silica beads (Biospec Products, Bartlesville, OK, USA), and DNA contamination was removed with the RNase-free DNase kit (Qiagen, Valencia, CA, USA) following the manufacturer’s protocol. 2 μL of total RNA from each sample was reverse transcribed to complementary DNA (cDNA) with the SuperScript III First Strand Synthesis System (Invitrogen, Carlsbad, CA, USA), following the manufacturer’s protocol with random hexamer priming. Template- and enzyme-free negative control reactions were performed in parallel.

From the sludge inocula, genomic DNA (gDNA) was extracted from two pooled replicate 250 mg samples with the PowerSoil DNA Extraction Kit (MoBio Laboratories, Carlsbad, CA, USA), following the manufacturer’s protocol with additional mechanical lysis as above. Sludge inoculum gDNA was used for bacterial community analysis only; the archaeal community composition has been previously reported (Wilkins et al., 2015). DNA concentration and purity was assessed with a NanoDrop 2000 UV-Vis Spectrophotometer (NanoDrop Products, Wilmington, DE, USA).

The transcribed bacterial V1–V3 and archaeal V1–V2 16S rRNA regions were amplified from template cDNA and gDNA (bacterial only) with the 27F/534R (Wu et al., 2010) and A2Fa/A571R (Kan et al., 2011) primer pairs respectively, with PCR ingredients and conditions per the cited studies for 30 amplification cycles in triplicate reactions. To enable multiplexed 454 pyrosequencing, barcode sequences were incorporated between the adaptors and forward primers (Hamady et al., 2008). Amplicons were pooled and purified with the Agencourt AMPure XP kit (Beckman Coulter, Pasadena, IN, USA), then quantified with the Quant-iT Broad-Range DNA Assay kit (Life Technologies, Grand Island, NY, USA). Equimolar concentrations from each sample were sequenced by BGI (Hong Kong sequencing facility) on a 454 GS FLX Titanium platform (Roche, Branchburg, NJ, USA). Pyrosequencing reads generated for this study have been deposited in the NCBI Sequence Read Archive under project # PRJNA275176.

Operational taxonomic units (OTUs) were generated for each domain separately following the UPARSE pipeline (Edgar, 2013), with culture cDNA and sludge inoculum gDNA samples pooled to aid direct taxonomic comparison. Demultiplexed reads were filtered to a maximum expected error of one error per read and trimmed to a uniform length of 122 bp using the “fastq_filter” command of USEARCH (version 7.0.109; Edgar, 2010). This length was selected to maximize sequence length while reducing the expected error rate to less than one error per read. Reads shorter than 122 bp were removed. Dereplicated reads were clustered using the “cluster_otus” command of USEARCH with the default radius of 0.03 (97% sequence similarity). Reads were assigned to OTUs using the “usearch_global” command of USEARCH. Each OTU was assigned a taxonomic lineage using the QIIME (version 1.8.0; Caporaso et al., 2010b) script “assign_taxonomy.py,” with the Greengenes (version 13_5; DeSantis et al., 2006) 97% similar 16S rRNA core reference set and taxonomy as reference. Due to the short sequence length, only taxonomic assignments to the genus level were considered. OTU representative sequences were aligned with PyNAST (Caporaso et al., 2010a) using the QIIME script “parallel_align_seqs_pynast.py” against the aligned Greengenes core reference set, and a tree built with FastTree (Price et al., 2010) using the QIIME script “make_phylogeny.py.” Chimeric OTU representative sequences were identified using the USEARCH command “uchime_ref” and the “Gold” database (http://drive5.com/uchime/gold.fa, retrieved 27 October 2014). Reads matching any of the following conditions were removed from downstream analysis: failed to cluster with at least one other read (singleton or failed to be assigned to OTU); belonged to OTU identified as chimeric; belonged to OTU with representative sequence that failed to align with PyNAST.

### Alpha and Beta Diversity

Inoculum DNA samples were excluded from all diversity analyses except for the construction of rarefaction curves, as differences in copy number between the DNA and RNA molecules would make direct comparison unreliable. Rarefaction curves were constructed to compare sample richness and assess whether richness was sampled to exhaustion. For each domain, 20 depths were selected at even intervals between one and the maximum sample read count for that domain. Each sample was randomly subsampled to each depth 10 times using the “rrarefy” function from the R package vegan (Oksanen et al., 2015), and the average OTU count at each depth calculated. To allow direct comparison of sample diversity, samples within each domain (excluding inoculum DNA samples) were randomly rarefied 10 times to the read count of the most depauperate sample in that domain. The number of OTUs, Chao1 richness estimator and abundance-based coverage estimator (ACE) were then calculated for each sample using the “estimateR” function from vegan and averaged. For each domain, the weighted UniFrac distance (Lozupone and Knight, 2005) between samples (excluding inoculum DNA samples) was calculated and the distances visualized with principal coordinates analysis (PCoA). To test if there were significant differences between the communities from different digesters and incubated with different substrates, analysis of similarities (ANOSIM) tests were performed using the “anosim” function from the vegan package. To test the hypothesis that the culture bacterial communities would be affected by the composition of the sludge inoculum, the combined culture (RNA) and inoculum (gDNA) OTU table was randomly subsampled to the depth of the most depauperate sample using “rrarefy” and the unweighted UniFrac distance calculated. The unweighted distance was selected in this case to minimize potential biases in copy number while comparing OTUs generated from both genomic and transcribed rRNA. The UniFrac distances between each culture sample and its source inoculum were compared to the distances between culture samples and the non-source inoculum, and statistical significance determined with the non-parametric Mann–Whitney test.

## Results

### Sequencing and OTU Formation

A total of 40,596 archaeal and 51,766 bacterial (including inoculum gDNA) 16S rRNA reads were obtained. Following read quality control, 34,595 archaeal and 39,550 bacterial reads were retained. Following OTU formation and quality control, 327 archaeal and 679 bacterial OTUs were formed comprising 28,020 and 24,802 reads respectively.

Because the bacterial and archaeal communities were assessed with different primer pairs and sequencing targets, richness and diversity comparisons were performed between samples and groups of samples but not between domains. No sample reached a richness plateau under rarefaction (Supplementary Figure S1), suggesting the OTU richness was not sampled to exhaustion. However, in both digesters and for both microbial domains, the rarefaction curve for the 55°C cellulose samples fell far below those for all other incubation conditions, suggesting these cultures had unusually low richness. When the samples were rarefied to equal depth (**Table 1**), 55°C cellulose samples were consistently the least rich in both OTU count and estimated richness (Chao1 and ACE). In samples inoculated from the GZ digester, samples amended with other substrates had similar observed and estimated richness within each domain (130–148 archaeal OTUs, 123–156 bacterial). However, SWH sludge amended with xylan had substantially higher observed and estimated richness (145 archaeal and 172 bacterial OTUs) than other SWH samples.

**Table 1:** List of cultures prepared for this study, methane and volatile fatty acid (VFA) production, and details on OTU formation and alpha diversity.

Digester	Condition	Methane (mL/g) (*SD*)^a^	Acetate (mM) (*SD*)^b^	Butyrate (mM) (*SD*)^b^	Propionate (mM) (*SD*)^b^	Domain	Reads^c^	OTUs^d^	Chao1^d^	ACE^d^
GZ	Cellulose	481.5 (71)	0 (0)	0 (0)	0 (0)	Archaea	2,479	148	192	204
						Bacteria	1,707	144	230	241
	Cellulose (55°C)	315 (24)	0.39 (0.01)	3.2 (0.07)	1.7 (0.42)	Archaea	3,438	45	54	57
						Bacteria	2,365	29	39	47
	Food waste	583.5 (6.4)	0 (0)	0 (0)	0 (0)	Archaea	3,672	132	200	192
						Bacteria	2,008	156	239	250
	Xylan	296 (16)	0 (0)	0 (0)	0 (0)	Archaea	3,271	138	172	173
						Bacteria	1,858	123	190	202
	Xylose	264 (47)	0 (0)	0 (0)	0 (0)	Archaea	3,025	130	168	170
						Bacteria	1,141	129	168	179
SWH	Cellulose	489.5 (77)	1.3 (0.01)	4.3 (0.52)	0 (0)	Archaea	3,045	74	110	113
						Bacteria	3,506	59	94	106
	Cellulose (55°C)	410 (28)	0.86 (0)	3.4 (0.2)	0.82 (0.23)	Archaea	2,318	34	36	36
						Bacteria	837	23	29	25
	Food waste	576 (31)	1.6 (0.13)	7.9 (0.21)	0.4 (0.14)	Archaea	2,434	60	73	76
						Bacteria	2,995	63	102	105
	Xylan	381 (25)	0 (0)	0 (0)	0 (0)	Archaea	2,070	145	194	204
						Bacteria	1,823	172	250	266
	Xylose	276 (28)	0 (0)	0 (0)	0 (0)	Archaea	2,268	90	109	116
						Bacteria	4,164	50	89	91

*^a^Average of replicate measurements of total production. ^b^Average of replicate measurements of final accumulated concentration. ^c^Quality-controlled reads contributing to the final OTU table. ^d^Averaged over 10 rounds of random subsampling to the depth of the most depauperate sample within each domain.*

### Taxonomic Composition

**FIGURE 1:**
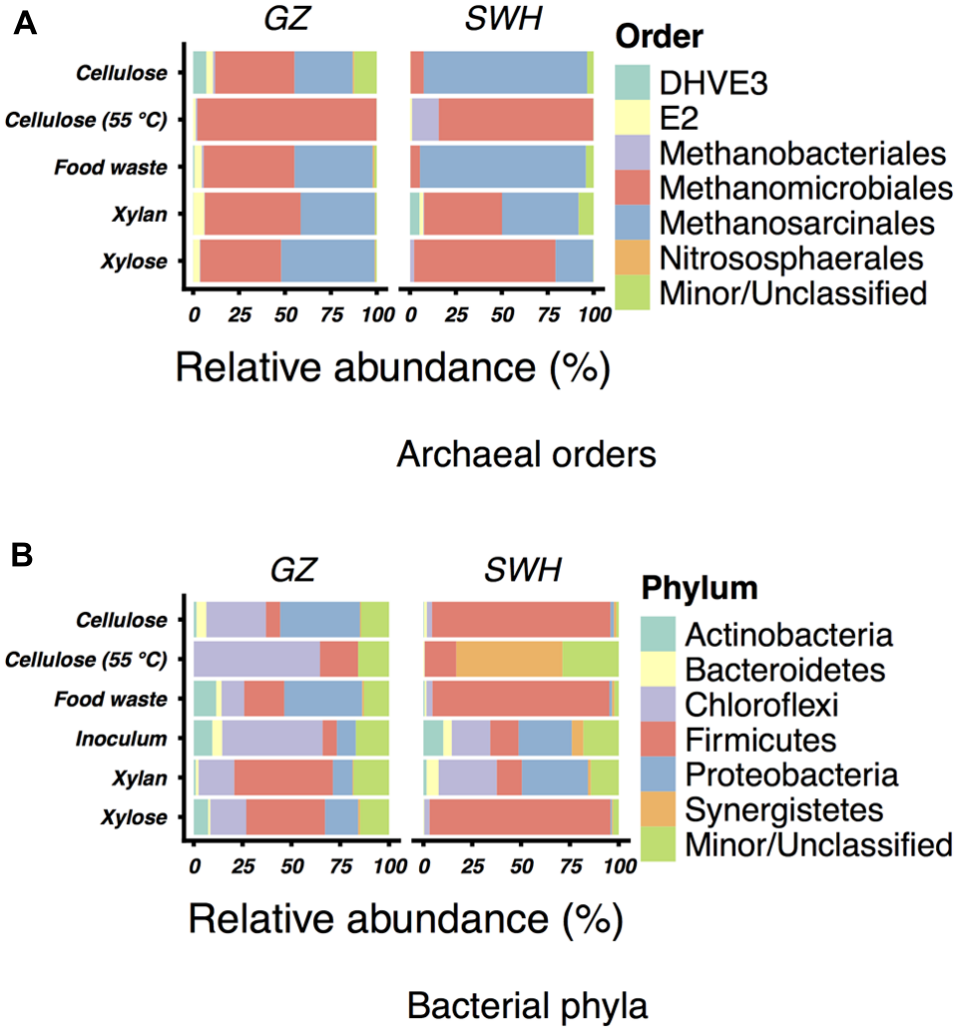
**Relative abundances of **(A)** archaeal orders in the enrichment cultures and **(B)** bacterial phyla in the cultures and sludge inocula.** For each, the six most abundant taxa (excluding unclassified OTUs) by mean relative abundance across all samples are shown. All less abundant and unclassified taxa are grouped in “Minor/Unclassified.” Abundances are for the complete (non-normalized) read set for each sample. Note that the Greengenes taxonomy includes some candidate and uncultured divisions.

Operational taxonomic units identified in this study were generally well-taxonomically classified, with 155 GZ archaea OTUs (66% of reads), 170 SWH archaea OTUs (74%), 105 GZ bacteria OTUs (37%), and 122 SWH bacteria OTUs (61%) classified to at least the genus level. At the phylum level, the archaeal community was consistently dominated by the *Euryarchaeota* (relative abundance 87–100%), with 0–1.2% *Crenarchaeota* and 0–11% unclassified. The two methanogenic orders *Methanomicrobiales* and *Methanosarcinales* dominated all cultures at the order level (**Figure 1**), with most containing at least 10–30% of each order.

All GZ incubations included a relatively high abundance of *Methanolinea* (23–93%), while *Methanolinea* was only abundant in the SWH xylan incubation (22%; **Table 2**). *Methanosaeta* were also quite abundant in GZ incubations (0–37%), with the major exception being cellulose at 55°C (0%). In contrast, *Methanoculleus* was present in GZ incubations at ≤0.16% but in SWH at 1.5–84%. *Methanosarcina* were present in SWH at 0.043–68% but in GZ at 0.058–24%.

**Table 2:** Relative abundances of the most abundant archaeal genera detected in this study.

	GZ					SWH				
Genus	Cellulose	Cellulose (55°C)	Food waste	Xylan	Xylose	Cellulose	Cellulose (55°C)	Food waste	Xylan	Xylose
*Methanoculleus*	0.16	0.058	—	—	—	5.1	84	3.5	1.5	61
*Methanosarcina*	0.4	0.058	0.84	0.52	24	55	0.043	68	1.1	13
*Methanothermobacter*	0.04	0.058	—	—	—	—	8.2	—	—	—
*Methanolinea*	23	93	25	32	24	0.36	—	0.62	22	4.8
*Methanosaeta*	29	—	37	24	5.4	0.56	—	0.041	28	7.2
*Methanobacterium*	0.16	0.76	0.16	0.12	0.099	0.033	—	0.082	—	2.2

*All genera with a relative abundance of at least 1% in one sample are included. Genera are given in descending order of their mean relative abundance across all samples. A dash indicates that no OTUs associated with the genus were detected. Note that the Greengenes taxonomy includes some candidate and uncultured divisions.*

Among the bacteria, the phyla *Firmicutes* (7.3–93%), *Chloroflexi* (0.12–65%), *Proteobacteria* (0–41%), and *Synergistetes* (0–54%) were most abundant (**Figure 1**). The sludge inocula bacterial communities from each digester were likewise dominated by the *Chloroflexi* (GZ) and *Chloroflexi*/*Firmicutes*/*Proteobacteria* (SWH), typical for AD sludge (Nelson et al., 2011). OTUs of the genus *Clostridium* (0.12–73%) were ubiquitous and abundant in all cultures but the SWH 55°C cellulose (0.12%), and were not abundant in the inoculum sludge (GZ 0.94%, SWH 0.57%). As with the archaeal genera, the 55°C cellulose samples contained a high abundance of genera particular to those samples. *Anaerobaculum*, while found in only two samples, comprised 54% of the SWH 55°C cellulose community. *Thermacetogenium*, likewise found in only two samples, was nevertheless 8.8% of the GZ 55°C cellulose community. Both of these idiosyncratic genera were absent from the sludge inocula (**Table 3**).

**Table 3:** Relative abundances of the most abundant bacterial genera detected in this study.

	GZ						SWH					
Genus	Inoculum	Cellulose	Cellulose (55°C)	Food waste	Xylan	Xylose	Inoculum	Cellulose	Cellulose (55°C)	Food waste	Xylan	Xylose
*Anaerobaculum*	—	—	—	—	—	—	—	—	54	—	0.11	—
*Geobacter*	2.7	19	—	12	5.0	6.8	—	—	—	—	17	0.024
*Thermacetogenium*	—	—	8.8	—	—	—	—	—	0.36	—	—	—
*Clostridium*	0.94	2.4	8.5	5.6	43	22	0.57	55	0.12	54	3.1	73
*Coprothermobacter*	—	—	—	—	—	—	—	—	4.7	—	—	—
*Desulfovibrio*	0.085	2.6	—	2.4	—	2.8	—	0.8	—	0.37	1.9	—
*Limnohabitans*	—	—	—	—	—	—	1.1	—	—	—	—	—
T78	0.26	—	—	—	—	—	10	2.1	—	2.5	5.3	2.2
vadinCA02	—	—	—	—	—	0.35	4.4	0.17	—	0.3	0.33	0.14
SHD-231	0.17	—	—	—	—	—	3.9	0.43	—	0.43	0.16	0.41
*Ethanoligenens*	2.0	—	—	0.15	—	0.088	—	—	—	—	—	—
*Pelotomaculum*	0.43	0.18	—	0.2	0.22	3.0	0.16	—	—	—	0.22	—
*Rhodobacter*	—	—	—	—	—	—	1.2	—	—	—	—	—
*Syntrophobacter*	2.0	13	—	12	3.0	4.6	—	0.31	—	0.27	11	0.072
*Syntrophomonas*	1.8	0.94	—	1.5	1.3	8.0	0.73	0.086	—	2.0	0.77	0.12
*Kosmotoga*	0.77	1.1	0.3	0.6	2.6	3.7	—	—	—	—	0.88	—
*Syntrophus*	0.34	1.0	—	3.2	0.27	0.44	0.082	0.057	—	—	0.44	0.58
*Treponema*	0.43	0.059	—	0.2	0.32	—	2.0	0.057	—	—	0.33	0.22

*All genera with a relative abundance of at least 1% in one sample are included. Genera are given in descending order of their mean relative abundance across all samples. A dash indicates that no OTUs associated with the genus were detected. Note that the Greengenes taxonomy includes some candidate and uncultured divisions.*

### Effect of Inoculum Source and Organic Waste Substrate

**FIGURE 2:**
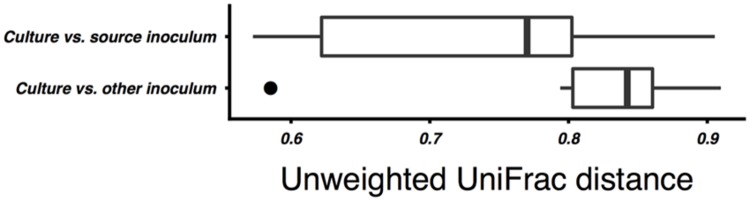
**Comparison of unweighted UniFrac distances between the bacterial communities of culture samples and the community of their source inoculum sludge vs. the non-source sludge.** Unweighted UniFrac distances were calculated on OTU counts subsampled to even depth. The difference between groups was statistically significant (Mann–Whitney, *p* < 0.05).

**FIGURE 3:**
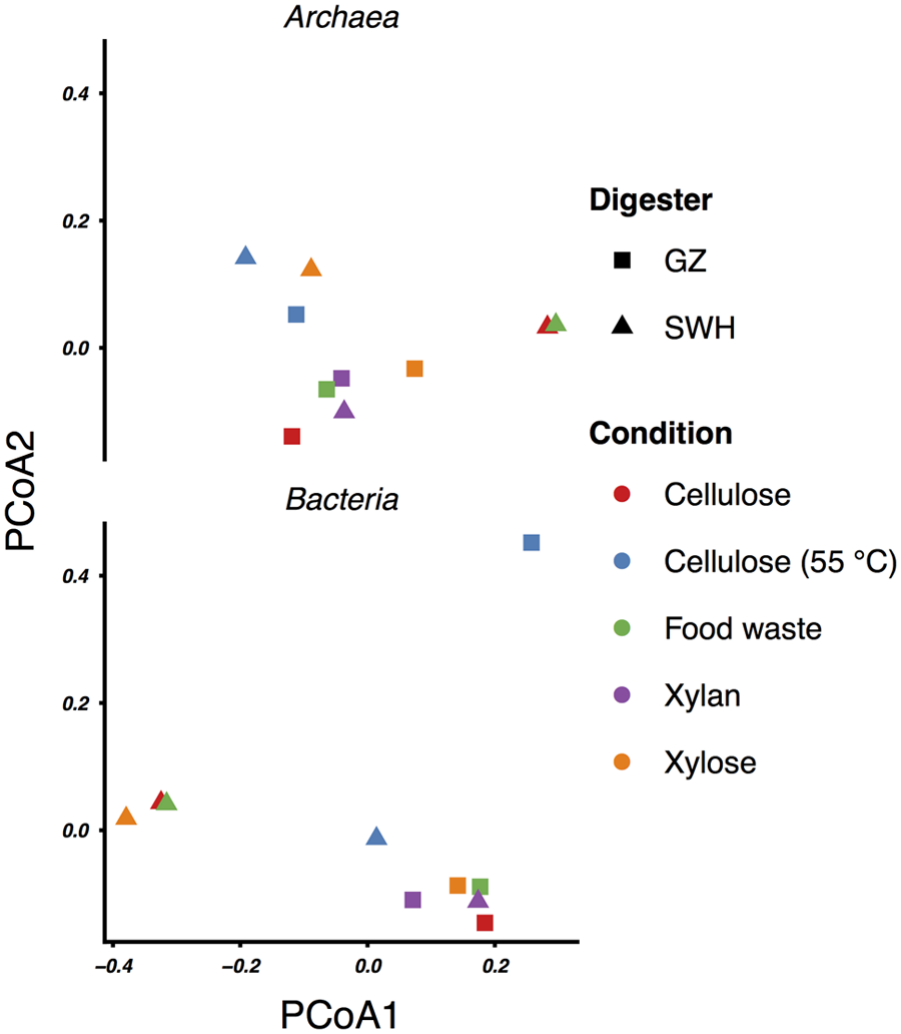
**Principal coordinates analysis (PCoA) ordinations of the weighted UniFrac distances between the archaeal and bacterial communities**.

On average, the enrichment bacterial communities resembled their respective source inoculm more closely (smaller unweighted UniFrac distance) than the other inoculum (Mann–Whitney p = 0.01, **Figure 2**). However, there was no statistically significant clustering of culture communities by either source digester or substrate (ANOSIM, all *p* ≥ 0.05; **Figure 3**). Because no significant community-level differences were identified for these two factors, we did not examine individual taxa for significant differences.

In the GZ cultures, the measured VFA (acetate, propionate, and butyrate) were only detected for cellulose amended cultures at 55°C, while in the SWH cultures they were detected for cultures amended with food waste, cellulose, and cellulose at 55°C (**Table 1**). Butyrate was the major VFA to accumulate in the cultures, reaching its highest concentration (7.9 mM) in the SWH food waste culture, which also had moderate concentrations of acetate and propionate (**Table 1**). By contrast, all three measured VFAs were below the detection threshold in the GZ food waste culture.

Food waste amendment resulted in the highest total methane productivity in batch cultures from both inoculum sources, with yield consistently varying in the order food waste > cellulose > cellulose (55°C) > xylan > xylose (**Table 1**). Methane yields for the same substrate but different inocula were within 1 standard deviation of each other, with the exception of cellulose (55°C) and xylan; in both cases the yield from the SWH culture was higher (**Table 1**). Negligible (<10 mL total) methane was produced from the control (without substrate) cultures.

## Discussion

This study aimed to characterize the microbial communities involved in digestion of waste substrates including food waste as well as lignocellulosic pre-treatment products. By reverse transcribing and sequencing 16S rRNA transcripts rather than the 16S rRNA gene (as is more typical in microbial community studies of the AD process (Nelson et al., 2011)), we were able to examine the active microbial populations. This is especially useful in closed systems such as laboratory cultures where cells from the inocula may persist despite being metabolically inactive (Lu et al., 2013), helping to reveal active members of complex communities that may otherwise be obscured in DNA-based surveys (Brettar et al., 2012).

### Methanogen Composition

Our previous study of the GZ and SWH sludge archaeal communities found on the basis of sequencing the methanogen-specific methyl coenzyme M reductase (*mcrA*) gene that the GZ sludge was *Methanomicrobiales*-dominated while SWH was *Methanosarcinales*-dominated (Wilkins et al., 2015). This study confirmed the high abundance of the *Methanomicrobiales* and *Methanosarcinales* (**Figure 1**), although the lack of a single dominant order in cultures inoculated from the same source suggests that substrate amendment disrupted any initial numerical advantage. Previous 16S rRNA gene sequencing of GZ and SWH sludge also revealed a substantial population of the uncultured ArcI/WSA2 group (Wilkins et al., 2015). The uncultured ArcI/WSA2 group of *Euryarchaeota*, frequently reported at high abundance in 16S rRNA gene-based AD community surveys (Chouari et al., 2005b; Rivière et al., 2009; Nelson et al., 2011; Wilkins et al., 2015), was conspicuous for its near-absence in this study, found at only 0–0.9% relative abundance across all samples. As this study targeted rRNA transcripts from active populations, it is possible that WSA2 were present but inactive. However, the WSA2 group are believed to be active methanogens, having been observed to grow in culture on formate and H_2_/CO_2_ (Chouari et al., 2005a) and possibly compete with *Methanosaeta* for acetate (Rivière et al., 2009). It is thus more likely that they are not a major component, active or otherwise, of the cultures.

### Effect of Inoculum Source and Organic Waste Substrate

Although the enrichment cultures’ bacterial communities were significantly more similar to their inoculum than to the other sludge sample, there was no significant clustering of communities by inoculum source or by substrate. While this may in part be attributable to the small sample size, it does suggest that inoculum source is not the major factor structuring the enriched communities. Despite these differences, all combinations of inoculum source and substrate resulted in methane-yielding communities.

As previously reported (Lu et al., 2013), food waste amendment resulted in the highest methane productivity in batch cultures from both inoculum sources, with yield consistently varying in the order food waste > cellulose > cellulose (55°C) > xylan > xylose (**Table 1**). The probable mechanism for the improved methane productivity of food waste-amended sewage sludge is that it reduces the protein concentration relative to carbohydrates and lipids, which are more labile under microbial hydrolysis, thereby increasing the microbial growth rate and the overall hydrolytic efficiency of the AD system (Iacovidou et al., 2012); the initial hydrolysis step is likely rate-limiting in the digestion of sewage sludge. Additionally, food waste may contain a higher proportion of available organic substrates than indicated by volatile solids analysis, for example in the form of VFAs or alcohols.

The SWH food waste-amended culture was also notable for its high accumulation of VFA, particularly butyrate (**Table 1**). In addition to fermentation from glucose, butyrate (and longer carboxylates) can be produced in AD systems by chain elongation of ethanol or lactate with acetate via the reverse β oxidation pathway, found in many AD bacteria (Spirito et al., 2014) including *Clostridium* sp. (Seedorf et al., 2008). This may account for the high accumulation of butyrate relative to acetate, which would be consumed, and to propionate, which can also be elongated by this route (Spirito et al., 2014). However, it is noteworthy that a similar accumulation pattern was observed in both 55°C cellulose cultures (**Table 1**); as the chain elongation pathway has been reported to be suppressed at this temperature in AD reactors (Spirito et al., 2014), it is unlikely that the butyrate was produced in these cultures by the same route. Rapid initial acidification and VFA production is typical in complex organic waste digestion, but is usually followed by acetogenic uptake and consequent buffering (Sosnowski et al., 2008). VFA accumulation may therefore indicate that VFA production is outstripping acetogenesis and potentially hindering digestion efficiency with the consequent pH decrease (Siegert and Banks, 2005; Iacovidou et al., 2012). In this case, however, the high butyrate concentrations do not seem to have hindered the efficiency of the SWH food waste culture, which had comparable methane production to the GZ culture (∼580 mL/g) despite the striking difference in VFA concentration.

The bacterial community in the SWH food waste culture was dominated by the genus *Clostridium* (54%), which was present but at much lower abundance in the GZ culture (5.6%). The dominant *Clostridium* OTU in the SWH food waste sample, OTU 1873 (52%; Supplementary Table S1), was not classified to the species level. Regardless, *Clostridium* species are common components of AD communities (Nelson et al., 2011) able to hydrolyze a diverse range of organic compounds, and produce metabolites including butyrate, acetate, and propionate (Tracy et al., 2012). The high abundance of *Clostridium* in the SWH food waste culture relative to VFA-consuming acetogens such as *Syntrophobacter* (GZ: 12%, SWH: 0.27%) and *Syntrophomonas* (GZ: 1.5%, SWH: 2%; Appels et al., 2008) thus suggests that *Clostridium*-driven VFA production was indeed proceeding faster than uptake in that sample. This may reflect differences in the inoculum microbial community, although *Clostridium* was quite abundant in other GZ cultures (e.g., 43% in GZ, 3.1% in SWH xylan cultures) and OTU 1873 was not detected in the SWH sludge inoculum (Supplementary Table S1). It may rather reflect the differing waste streams treated by the two source digesters, with the sugar-rich beverage factory waste stream treated by the GZ digester providing a higher concentration of labile carbohydrates for hydrolysis and/or fermentation.

Cellulose was the second most efficient organic substrate, with the mesophilic (35°C) culture outperforming the thermophilic (55°C; **Table 1**). Temperature affects several aspects of AD performance, including the microbial growth rate, inhibition of certain community members, enzyme kinetics and the solubility of organic substrates and intermediate compounds (Appels et al., 2008). As both source digesters were operated at mesophilic temperatures, it is likely that the sludge inocula communities were adapted to mesophilic conditions. The most obvious effect of thermophilic growth on the microbial community was the much lower bacterial and archaeal richness relative to the mesophilic cultures (**Table 1**), although this did not result in a convergence of the community profiles (**Figure 3**). Of the archaea, the GZ thermophilic cellulose community was dominated by the genus *Methanolinea* (93%) while the SWH culture was dominated by *Methanoculleus* (84%), neither of which were major components of AD sludge from either digester (Wilkins et al., 2015). Both the recently described genus *Methanolinea* (Imachi et al., 2008) and members of the *Methanoculleus* (Barret et al., 2013) perform methanogenesis via the hydrogenotrophic pathway, which can be coupled with syntrophic bacterial oxidation of VFAs to H_2_/CO_2_. The bacterial communities were also dominated by a small number of OTUs. GZ was dominated by two OTUs from the poorly described phylum *Chloroflexi*, OTU 5 (family *Anaerolinaceae*, 45%) and OTU 9 (class *Anaerolineae*, 20%). The *Chloroflexi* are frequently found in high abundance in AD communities (Björnsson et al., 2002; Nelson et al., 2011). While their role in AD systems remains poorly described (Rivière et al., 2009; Narihiro et al., 2015), isolates from AD reactors have been found to grow fermentatively (Yamada and Sekiguchi, 2009 and references therein) and some exhibit faster growth when co-cultivated with hydrogenotrophic methanogens (Yamada et al., 2005, 2007), suggesting a syntrophic or “semi-syntrophic” (Yamada and Sekiguchi, 2009) role. Similarly, OTU 10 (genus *Anaerobaculum*) comprised 53% of all bacteria in the SWH sample and belongs to a group (the *Synergistetes*) also identified as a “core” taxon in AD communities (Rivière et al., 2009) but with an unknown role (Narihiro et al., 2015), although some *Synergistetes* have been shown to utilize acetate and are likely syntrophically coupled with hydrogenotrophic methanogens (Ito et al., 2011). If it is assumed that these dominant bacteria are indeed syntrophic partners of the hydrogenotrophic methanogens found in both cultures, these low-richness communities represent assembly of phylogenetically distant OTUs to perform nevertheless similar roles in the AD process, and resulting in similar methane productivity (**Table 1**). Given that all of these OTUs were undetected or present at <1% in the respective source digester’s sludge inoculum (Supplementary Table S1), it is likely that they were inactive and/or at very low abundance in the inoculum and increased in abundance due to cellulose amendment and adaptation to thermophilic growth.

While the mesophilic (35°C) cellulose cultures were overall richer than the thermophilic (**Table 1**), these communities were also dominated by a relatively small number of OTUs in the SWH culture, although the GZ culture was relatively diverse. While the genera *Methanosaeta* (29%) and *Methanolinea* (23%) comprised the majority of the GZ archaea, these genera were each represented by a number of OTUs, and several OTUs classified only to the family *Methanoregulaceae* were also present at 1–10% (Supplementary Table S1). By contrast, the archaeal community in the SWH mesophilic cellulose culture was dominated by two OTUs classified to the order *Methanosarcinales*, OTU 3 (43%) and OTU 4 (30%). Overall, the SWH methanogen community was dominated by the acetoclastic order *Methanosarcinales*, while the GZ culture contained a more even mix of *Methanosarcinales* and hydrogenotrophic *Methanomicrobiales*, a similar compositional pattern to that observed for food waste (**Figure 1**) and consistent with the overall similarities between the mesophilic cellulose and food waste communities for each digester (**Figure 3**). This again suggests that the composition of the sludge inoculum within a digestion culture has a large effect on the relative contribution of the acetoclastic and hydrogenotrophic pathways in the active community, even with the substrate composition held constant. This is consistent with the demonstrated similarity of culture bacterial communities to their respective sludge inocula (**Figure 2**).

Cultures amended with xylan, the least efficient methane producers (**Table 1**) as previously reported (Lu et al., 2013), and with xylose also had similar archaeal and bacterial communities within each sludge source (with the exception of SWH bacteria; **Figures 1** and **3**). For both substrates and source digesters, the methanogen community contained substantial populations of both *Methanosarcinales* and *Methanomicrobiales* while the bacterial communities were dominated by the *Firmicutes, Chloroflexi* and *Proteobacteria* (**Figure 1**). The SWH xylan cultures were notable for their unusually high observed and estimated total richness (**Table 1**). This cannot be attributed to noise introduced during PCR or sequencing, as it was independently observed in the (separately sequenced) archaeal and bacterial communities and as the number of unique OTUs in this sample was not unusually high, indicating that they were simply enriched in OTUs also found in other samples. Compared to simpler and monomeric substrates (e.g., xylose), xylan also requires a large set of enzymes (xylanases) for complete hydrolysis (Pérez et al., 2002) and consequently produces a broad range of fermentation substrates. The higher richness may therefore reflect the presence in the SWH sludge inoculum of taxa able to utilize xylan and its hydrolysis products, which were subsequently enriched.

While AD of xylose to methane has been little studied, Temudo et al. (2008) examined biomass production from anaerobic sludge inocula grown on either glucose or xylose as the sole carbon source. They reported that xylose resulted in 20% lower biomass yield than glucose, and suggested that the higher ATP requirement for xylose active transport in bacteria resulted in an lower net energetic yield for xylose than glucose per mole of substrate (Temudo et al., 2008) assuming that xylose transport in the relevant species is effected by high-affinity ABC transporters, while glucose transport proceeds mainly via an ATP-independent route such as the phosphotransferase system (PTS). As at least some anaerobic bacteria possess ATP-independent uptake systems for cellobiose (Kajikawa and Masaki, 1999), the major hydrolysis product of cellulose [e.g., when hydrolyzed by extracellular cellulosomes such as that produced by *Clostridium thermocellum* (Bégum and Lemaire, 1996)], cellulose may enjoy a similar energetic advantage over xylose that would account for the difference in methane yield.

This study aimed to characterize the effects of differences in sludge inocula and organic substrate on the microbial communities associated with AD and their methane yield. Narihiro et al. (2015) reported a similar investigation in which anaerobic digester sludge and swine manure were amended with a range of intermediate AD substrates including acetate and fatty acids, and found that the resulting enriched bacterial and archaeal communities clustered significantly by substrate, with acetate-amended communities clustering further by inoculum source. Similarly, a meta-analysis of 16S rRNA gene clones from AD reactors (Zhang et al., 2014) and denaturing gradient gel electrophoresis analysis of organic waste AD reactors (Regueiro et al., 2013) found that communities clustered by substrate. In this study, however, while there were some similarities by inoculum source and substrate in the active community taxonomic compositions (e.g., the presence or absence of certain genera; **Tables 2** and **3**), ANOSIM did not support a statistically significant grouping of either the archaeal or bacterial communities by source digester or growth substrate although bacterial communities were on average more similar to their source inoculum sludge than to the non-source sludge (**Figure 2**). Given that methane yields were relatively consistent across substrates (**Table 1**), this suggests that microbial communities assembled from different inocula to perform similar digestion tasks may have similar efficiency despite differing composition. We plan to confirm these results with a larger set of source inocula, which will also provide additional statistical power to test clustering of the communities by inoculum source and substrate. Additional experiments are also needed to better characterize the microbial communities, including by sequencing the active populations in the initial inoculum and comparing the active to dormant populations by parallel sequencing of 16S rRNA genes, and by culturing in a continuous flow system using pre-treated lignocellulosic matter as a substrate to better simulate a practical reactor. Tag pyrosequencing of the methanogen-specific *mcrA* gene (Wilkins et al., 2015) would also be useful to better characterize the as yet uncultivated fraction of the methanogen population.

## Author Contributions

DW and SR analyzed data and wrote the manuscript; XL performed laboratory work; PL designed the experiment and wrote the manuscript.

## Conflict of Interest Statement

The authors declare that the research was conducted in the absence of any commercial or financial relationships that could be construed as a potential conflict of interest.
