# Supplementary material for: Effects of sludge inoculum and organic feedstock on active microbial communities and methane yield during anaerobic digestion
Source: Front Microbiol. 2015 Oct 13;6:1114. doi: 10.3389/fmicb.2015.01114 (PMC4602121; doi:10.3389/fmicb.2015.01114)
Supplement: Supplementary file 1 [file Table_1.PDF]

Table S1: Relative abundances of the most abundant OTUs detected in this study. For each domain, all OTUs with a relative abundance of at least 1% in one sample are included. OTUs are given in descending order of their mean relative abundance across all samples. Each OTU is preceded by the rank and taxon for the deepest rank to which that OTU was classified. Note that the Greengenes taxonomy includes some candidate and uncultured divisions.

		GZ						SWH					
	OTU	Inoculum	Cellulose	Cellulose (55 °C)	Food waste	Xylan	Xylose	Inoculum	Cellulose	Cellulose (55 °C)	Food waste	Xylan	Xylose
Archaea	Class: Methanomicrobia (OTU 288)		0.04	0.0	0.027	0.28	0.43		2.7	0.0	3.3	2.3	0.0
	Family: [Methanomassiliicoccaceae] (OTU 11)		1.6	0.0	1.4	2.4	1.5		0.0	0.0	0.041	0.63	0.0
	Family: [Methanomassiliicoccaceae] (OTU 16)		0.89	0.0	1.1	1.8	0.36		0.033	0.043	0.0	0.14	0.0
	Family: [Methanomassiliicoccaceae] (OTU 21)		0.16	1.0	0.082	0.28	0.099		0.033	0.0	0.082	0.43	0.088
	Family: Methanobacteriaceae (OTU 339)		0.04	0.0	0.0	0.0	0.0		0.0	3.8	0.0	0.0	0.0
	Family: Methanobacteriaceae (OTU 55)		0.0	0.058	0.0	0.0	0.0		0.0	2.0	0.0	0.0	0.0
	Family: Methanoregulaceae (OTU 257)		2.1	0.029	3.1	1.4	2.2		0.033	0.0	0.041	1.7	0.93
	Family: Methanoregulaceae (OTU 273)		3.2	0.029	3.9	3.6	3.7		0.13	0.0	0.0	2.9	0.0
	Family: Methanoregulaceae (OTU 5)		8.4	0.0	12	9.4	8.7		0.23	0.0	0.12	11	0.044
	Family: Methanoregulaceae (OTU 54)		1.1	0.058	0.98	0.89	1.4		0.0	0.0	0.0	0.63	0.044
	Family: Methanoregulaceae (OTU 588)		0.4	1.7	0.054	0.4	0.099		0.0	0.0	0.0	0.14	7.1
	Family: Methanoregulaceae (OTU 609)		0.81	1.4	0.65	0.76	1.2		0.0	0.0	0.0	0.43	0.18
	Family: Methanoregulaceae (OTU 95)		0.69	0.0	1.1	0.7	0.63		0.0	0.0	0.0	0.58	0.0
	Family: Methanosarcinaceae (OTU 410)		0.12	0.0	0.22	1.2	0.0		0.0	0.0	0.0	0.0	0.0
	Family: Methanosarcinaceae (OTU 437)		0.36	0.0	1.1	2.9	0.2		0.0	0.0	0.0	0.14	0.0
	Family: Methanosarcinaceae (OTU 9)		1.0	0.0	2.3	9.0	0.53		0.0	0.0	0.0	0.77	0.0
	Genus: *Methanobacterium* (OTU 28)		0.0	0.0	0.0	0.0	0.0		0.0	0.0	0.0	0.0	1.9
	Genus: *Methanoculleus* (OTU 12)		0.04	0.0	0.0	0.0	0.0		4.5	0.0	3.1	0.0	0.088
	Genus: *Methanoculleus* (OTU 153)		0.0	0.0	0.0	0.0	0.0		0.0	0.086	0.0	0.0	7.9
	Genus: *Methanoculleus* (OTU 2)		0.0	0.0	0.0	0.0	0.0		0.0	52	0.0	0.29	34
	Genus: *Methanoculleus* (OTU 27)		0.12	0.0	0.0	0.0	0.0		0.0	5.9	0.0	0.048	0.4
	Genus: *Methanoculleus* (OTU 307)		0.0	0.058	0.0	0.0	0.0		0.099	0.043	0.041	0.39	4.2
	Genus: *Methanoculleus* (OTU 325)		0.0	0.0	0.0	0.0	0.0		0.0	1.2	0.0	0.0	0.53
	Genus: *Methanoculleus* (OTU 340)		0.0	0.0	0.0	0.0	0.0		0.0	4.4	0.0	0.0	0.31
	Genus: *Methanoculleus* (OTU 534)		0.0	0.0	0.0	0.0	0.0		0.0	3.8	0.0	0.0	0.088
	Genus: *Methanoculleus* (OTU 683)		0.0	0.0	0.0	0.0	0.0		0.0	2.8	0.0	0.0	0.0
	Genus: *Methanoculleus* (OTU 712)		0.0	0.0	0.0	0.0	0.0		0.0	9.9	0.0	0.048	11
	Genus: *Methanoculleus* (OTU 77)		0.0	0.0	0.0	0.0	0.0		0.0	1.1	0.0	0.0	0.4
	Genus: *Methanolinea* (OTU 1)		0.48	67	0.3	0.83	0.5		0.066	0.0	0.082	0.63	0.0
	Genus: *Methanolinea* (OTU 123)		4.8	0.029	7.8	3.8	5.6		0.0	0.0	0.082	2.7	0.0
	Genus: *Methanolinea* (OTU 232)		0.93	0.0	0.84	0.95	0.86		0.0	0.0	0.0	1.2	0.0
	Genus: *Methanolinea* (OTU 235)		0.44	0.0	0.49	0.89	0.4		0.0	0.0	0.082	1.1	0.0
	Genus: *Methanolinea* (OTU 357)		6.0	0.67	6.9	6.1	6.9		0.033	0.0	0.0	3.5	0.0
	Genus: *Methanolinea* (OTU 456)		0.04	15	0.027	0.061	0.0		0.0	0.0	0.0	0.0	0.0
	Genus: *Methanolinea* (OTU 490)		1.1	6.7	0.65	2.0	1.0		0.033	0.0	0.0	1.5	3.4
	Genus: *Methanolinea* (OTU 583)		4.6	0.15	3.7	11	4.1		0.2	0.0	0.29	6.2	0.0
	Genus: *Methanolinea* (OTU 602)		1.8	0.17	1.1	1.4	0.76		0.033	0.0	0.0	1.2	0.044
	Genus: *Methanolinea* (OTU 675)		0.24	0.029	0.16	1.4	0.23		0.0	0.0	0.0	0.72	0.0
	Genus: *Methanolinea* (OTU 68)		0.04	1.0	0.027	0.031	0.033		0.0	0.0	0.0	0.0	0.0
	Genus: *Methanosaeta* (OTU 100)		1.3	0.0	1.8	0.67	0.033		0.033	0.0	0.0	2.1	0.0
	Genus: *Methanosaeta* (OTU 14)		0.93	0.0	1.7	2.6	0.99		0.0	0.0	0.0	1.3	0.0
	Genus: *Methanosaeta* (OTU 149)		1.2	0.0	1.3	0.76	0.0		0.033	0.0	0.0	1.4	0.0
	Genus: *Methanosaeta* (OTU 253)		4.6	0.0	4.2	2.5	0.2		0.0	0.0	0.0	2.4	0.0
	Genus: *Methanosaeta* (OTU 402)		1.9	0.0	2.6	0.95	0.76		0.033	0.0	0.0	1.1	0.044
	Genus: *Methanosaeta* (OTU 540)		1.2	0.0	0.98	0.43	0.066		0.0	0.0	0.0	0.77	0.0
	Genus: *Methanosaeta* (OTU 563)		1.0	0.0	1.2	0.55	0.23		0.0	0.0	0.0	0.87	5.5
	Genus: *Methanosaeta* (OTU 6)		8.5	0.0	9.3	6.1	0.3		0.13	0.0	0.0	8.1	0.0
	Genus: *Methanosaeta* (OTU 8)		2.3	0.0	6.3	3.0	1.3		0.13	0.0	0.0	3.3	0.26
	Genus: *Methanosarcina* (OTU 233)		0.0	0.029	0.0	0.0	0.0		1.6	0.0	2.8	0.048	4.9
	Genus: *Methanosarcina* (OTU 24)		0.0	0.0	0.0	0.0	0.0		0.26	0.043	0.58	0.0	3.0
	Genus: *Methanosarcina* (OTU 26)		0.0	0.0	0.0	0.0	0.0		1.1	0.0	0.7	0.0	0.0
	Genus: *Methanosarcina* (OTU 3)		0.0	0.029	0.054	0.031	0.73		43	0.0	49	0.34	1.4
	Genus: *Methanosarcina* (OTU 322)		0.0	0.0	0.0	0.0	0.0		0.56	0.0	1.4	0.0	0.0
	Genus: *Methanosarcina* (OTU 33)		0.04	0.0	0.0	0.031	0.99		1.1	0.0	1.4	0.048	0.0
	Genus: *Methanosarcina* (OTU 50)		0.0	0.0	0.0	0.0	0.0		0.76	0.0	1.1	0.0	0.0
	Genus: *Methanosarcina* (OTU 522)		0.0	0.0	0.0	0.0	0.89		2.2	0.0	1.6	0.048	0.4
	Genus: *Methanosarcina* (OTU 529)		0.0	0.0	0.0	0.0	0.066		2.2	0.0	5.3	0.0	0.0
	Genus: *Methanosarcina* (OTU 570)		0.24	0.0	0.54	0.28	11		0.033	0.0	0.041	0.097	0.0
	Genus: *Methanosarcina* (OTU 60)		0.0	0.0	0.027	0.0	1.7		0.3	0.0	0.33	0.0	0.0
	Genus: *Methanosarcina* (OTU 63)		0.04	0.0	0.14	0.12	2.4		0.16	0.0	0.29	0.29	0.044
	Genus: *Methanosarcina* (OTU 87)		0.081	0.0	0.027	0.061	1.8		0.033	0.0	0.082	0.048	0.0
	Methanothermobacter thermautotrophicus (OTU 333)		0.0	0.029	0.0	0.0	0.0		0.0	1.1	0.0	0.0	0.0
	Methanothermobacter thermautotrophicus (OTU 341)		0.04	0.0	0.0	0.0	0.0		0.0	6.1	0.0	0.0	0.0
	Order: DHVE3 (OTU 10)		7.1	0.0	0.68	0.15	0.066		0.0	0.0	0.0	5.2	0.0
	Order: Methanomicrobiales (OTU 37)		0.04	0.029	0.0	0.061	0.0		0.0	0.0	0.0	0.19	1.8
	Order: Methanosarcinales (OTU 242)		0.04	0.0	0.082	0.31	1.6		0.0	0.0	0.0	0.0	0.044
	Order: Methanosarcinales (OTU 274)		0.081	0.0	0.19	0.28	2.4		0.033	0.0	0.0	0.048	0.18
	Order: Methanosarcinales (OTU 4)		0.081	0.0	0.054	1.0	2.3		30	0.0	21	10	0.0
	Order: Methanosarcinales (OTU 404)		0.32	0.0	0.71	0.79	13		0.066	0.0	0.0	0.58	0.0
	Order: Methanosarcinales (OTU 659)		0.0	0.0	0.027	0.12	0.33		2.4	0.0	1.2	0.82	0.044
	Unknown (OTU 109)		5.2	0.0	0.35	0.21	0.033		0.033	0.0	0.0	2.9	0.0
	Unknown (OTU 120)		5.6	0.0	0.52	0.031	0.0		0.0	0.0	0.0	2.2	0.0
Bacteria	Class: Anaerolineae (OTU 124)	1.2	0.0	0.0	0.05	0.054	0.61	0.0	0.0	0.0	0.0	0.055	0.0
	Class: NPL-UPA2 (OTU 51)	0.0	1.6	0.0	0.55	0.75	0.0	0.0	0.0	0.0	0.0	0.66	0.0
	Clostridium ljungdahlii (OTU 313)	0.0	0.0	0.0	0.05	0.0	0.088	0.0	1.3	0.0	1.1	0.0	0.74
	Family: [Thermodesulfovibrionaceae] (OTU 60)	0.34	1.1	0.0	0.5	0.7	0.18	0.0	0.0	0.0	0.0	0.93	0.0
	Family: [Tissierellaceae] (OTU 204)	0.0	0.0	0.0	0.0	0.0	0.0	0.0	0.0	2.2	0.0	0.0	0.0
	Family: Anaerolinaceae (OTU 1596)	3.5	3.0	0.0	3.1	1.5	2.5	0.0	0.0	0.12	0.0	2.6	0.0
	Family: Anaerolinaceae (OTU 160)	1.5	0.29	0.0	0.2	0.0	0.26	0.0	0.0	0.0	0.0	0.16	0.0
	Family: Anaerolinaceae (OTU 196)	0.0	0.0	0.0	0.0	0.0	1.1	0.0	0.0	0.0	0.0	0.0	0.0
	Family: Anaerolinaceae (OTU 28)	10	0.88	0.0	1.4	0.32	1.5	0.0	0.0	0.0	0.0	0.66	0.0
	Family: Anaerolinaceae (OTU 5)	0.17	0.059	45	0.05	0.0	0.0	0.0	0.0	0.0	0.0	0.0	0.0
	Family: Anaerolinaceae (OTU 62)	1.5	0.12	0.0	0.9	0.27	1.3	0.0	0.0	0.0	0.0	0.27	0.0
	Family: Beijerinckiaceae (OTU 37)	0.0	0.0	0.0	0.0	0.0	0.0	7.4	0.14	0.0	0.067	0.0	0.0
	Family: Clostridiaceae (OTU 1)	0.0	0.12	0.042	0.5	0.0	0.7	0.0	8.0	0.0	8.3	0.33	11
	Family: Clostridiaceae (OTU 11)	0.0	0.0	0.0	0.2	0.0	0.0	0.41	7.5	0.0	7.2	0.33	0.41
	Family: Clostridiaceae (OTU 1167)	0.085	0.0	0.0	0.0	1.2	0.79	0.0	0.14	0.0	0.2	0.0	0.12
	Family: Clostridiaceae (OTU 1741)	0.0	0.0	0.0	0.1	2.5	2.1	0.0	0.0	0.0	0.0	0.0	0.0
	Family: Clostridiaceae (OTU 1903)	0.0	0.059	0.0	0.3	0.0	0.088	0.0	2.8	0.0	2.2	0.49	3.1
	Family: Clostridiaceae (OTU 29)	0.085	0.12	0.0	0.1	0.0	0.18	1.5	1.2	0.0	1.4	0.38	0.94
	Family: Clostridiaceae (OTU 593)	0.0	0.0	0.0	0.05	0.0	0.0	0.24	1.1	0.0	1.6	0.055	0.072
	Family: Clostridiaceae (OTU 8)	0.0	0.059	0.0	0.05	0.0	0.0	0.9	8.7	0.0	6.9	0.11	0.31
	Family: Gracilibacteraceae (OTU 194)	0.0	0.0	0.0	0.0	0.0	0.0	1.1	0.0	0.0	0.0	0.0	0.0
	Family: Nocardioidaceae (OTU 91)	0.0	0.0	0.0	0.0	0.0	0.0	2.4	0.0	0.0	0.0	0.0	0.0
	Family: Rhodocyclaceae (OTU 70)	0.085	0.059	0.0	1.6	0.0	0.0	0.49	0.0	0.0	0.0	0.11	0.0
	Family: Ruminococcaceae (OTU 122)	0.0	0.0	0.0	0.0	0.0	0.0	1.8	0.0	0.0	0.0	0.0	0.0
	Family: SHA-116 (OTU 137)	0.0	0.0	0.0	0.0	0.0	0.0	2.3	0.029	0.0	0.0	0.0	0.072
	Family: SHA-31 (OTU 9)	0.0	0.0	20	0.0	0.0	0.0	0.0	0.0	0.0	0.0	0.0	0.0
	Family: Syntrophaceae (OTU 23)	0.6	2.4	0.0	4.0	0.54	0.44	0.0	0.0	0.0	0.0	1.4	0.0
	Family: Syntrophorhabdaceae (OTU 77)	0.085	0.35	0.0	1.1	0.11	0.0	0.0	0.0	0.0	0.0	0.38	0.0
	Family: Syntrophorhabdaceae (OTU 83)	1.0	0.23	0.0	0.6	0.22	0.0	0.0	0.0	0.0	0.0	0.22	0.0
	Family: TIBD11 (OTU 32)	0.0	0.0	0.47	0.0	0.0	0.0	0.0	0.0	20	0.0	0.0	0.0
	Family: Veillonellaceae (OTU 66)	0.17	0.41	0.0	1.7	0.0	0.0	0.0	0.0	0.0	0.0	0.49	0.0
	Genus: *Anaerobaculum* (OTU 10)	0.0	0.0	0.0	0.0	0.0	0.0	0.0	0.0	53	0.0	0.055	0.0
	Genus: *Anaerobaculum* (OTU 1282)	0.0	0.0	0.0	0.0	0.0	0.0	0.0	0.0	1.4	0.0	0.055	0.0
	Genus: *Clostridium* (OTU 168)	0.0	0.0	0.0	0.15	0.0	1.1	0.0	0.0	0.0	0.0	0.055	0.0
	Genus: *Clostridium* (OTU 1743)	0.0	0.0	0.0	0.0	1.6	0.44	0.0	0.0	0.0	0.0	0.0	0.0
	Genus: *Clostridium* (OTU 1873)	0.0	0.059	0.042	1.3	0.0	1.2	0.0	53	0.0	52	1.2	71
	Genus: *Clostridium* (OTU 20)	0.0	0.0	8.4	0.0	0.0	0.0	0.0	0.0	0.0	0.0	0.0	0.0
	Genus: *Clostridium* (OTU 27)	0.43	2.2	0.0	2.3	1.5	2.2	0.0	0.0	0.0	0.0	0.82	0.0
	Genus: *Clostridium* (OTU 30)	0.0	0.0	0.0	0.25	8.0	0.0	0.0	0.0	0.0	0.0	0.11	0.0
	Genus: *Clostridium* (OTU 38)	0.0	0.0	0.0	0.0	7.1	0.0	0.0	0.0	0.0	0.0	0.38	0.0
	Genus: *Clostridium* (OTU 50)	0.0	0.0	0.0	0.2	6.4	0.88	0.0	0.0	0.0	0.0	0.0	0.048
	Genus: *Clostridium* (OTU 809)	0.0	0.0	0.0	0.55	15	13	0.0	0.0	0.0	0.0	0.33	0.048

		GZ						SWH					
	**OTU**	**Inoculum**	**Cellulose**	**Cellulose (55 °C)**	**Food waste**	**Xylan**	**Xylose**	**Inoculum**	**Cellulose**	**Cellulose (55 °C)**	**Food waste**	**Xylan**	**Xylose**
Bacteria (cont.)	Genus: *Clostridium* (OTU 819)	0.0	0.0	0.0	0.0	3.0	0.0	0.0	0.0	0.0	0.0	0.0	0.0
	Genus: *Clostridium* (OTU 98)	0.0	0.0	0.0	0.0	0.054	2.7	0.0	0.0	0.0	0.0	0.0	0.024
	Genus: *Coprothermobacter* (OTU 609)	0.0	0.0	0.0	0.0	0.0	0.0	0.0	0.0	3.8	0.0	0.0	0.0
	Genus: *Desulfovibrio* (OTU 42)	0.085	2.6	0.0	2.4	0.0	2.8	0.0	0.8	0.0	0.37	1.9	0.0
	Genus: *Ethanoligenens* (OTU 143)	2.0	0.0	0.0	0.15	0.0	0.088	0.0	0.0	0.0	0.0	0.0	0.0
	Genus: *Geobacter* (OTU 22)	0.085	2.3	0.0	2.1	1.6	3.6	0.0	0.0	0.0	0.0	1.9	0.024
	Genus: *Geobacter* (OTU 6)	2.6	17	0.0	10	3.4	3.2	0.0	0.0	0.0	0.0	15	0.0
	Genus: *Limnohabitans* (OTU 181)	0.0	0.0	0.0	0.0	0.0	0.0	1.1	0.0	0.0	0.0	0.0	0.0
	Genus: *Pelotomaculum* (OTU 58)	0.43	0.18	0.0	0.2	0.22	3.0	0.0	0.0	0.0	0.0	0.22	0.0
	Genus: *Rhodobacter* (OTU 193)	0.0	0.0	0.0	0.0	0.0	0.0	1.1	0.0	0.0	0.0	0.0	0.0
	Genus: SHD-231 (OTU 48)	0.0	0.0	0.0	0.0	0.0	0.0	1.6	0.43	0.0	0.43	0.16	0.41
	Genus: SHD-231 (OTU 95)	0.17	0.0	0.0	0.0	0.0	0.0	2.4	0.0	0.0	0.0	0.0	0.0
	Genus: *Syntrophobacter* (OTU 14)	1.6	8.4	0.0	7.1	1.8	2.3	0.0	0.0	0.0	0.0	6.7	0.0
	Genus: *Syntrophobacter* (OTU 959)	0.0	0.64	0.0	1.1	0.22	0.88	0.0	0.0	0.0	0.0	0.22	0.0
	Genus: *Syntrophomonas* (OTU 46)	0.85	0.59	0.0	0.55	0.7	3.2	0.0	0.0	0.0	0.0	0.27	0.0
	Genus: *Syntrophomonas* (OTU 68)	0.6	0.18	0.0	0.55	0.32	1.8	0.0	0.0	0.0	0.0	0.22	0.0
	Genus: *Syntrophomonas* (OTU 71)	0.0	0.0	0.0	0.0	0.0	0.0	0.0	0.057	0.0	1.3	0.0	0.072
	Genus: *Syntrophomonas* (OTU 90)	0.34	0.18	0.0	0.4	0.32	2.9	0.57	0.0	0.0	0.0	0.27	0.048
	Genus: *Syntrophus* (OTU 44)	0.34	0.59	0.0	3.1	0.22	0.26	0.0	0.0	0.0	0.0	0.27	0.048
	Genus: T78 (OTU 12)	0.0	0.0	0.0	0.0	0.0	0.0	8.7	1.9	0.0	2.1	4.2	2.1
	Genus: *Thermacetogenium* (OTU 39)	0.0	0.0	8.5	0.0	0.0	0.0	0.0	0.0	0.36	0.0	0.0	0.0
	Genus: *Treponema* (OTU 105)	0.0	0.0	0.0	0.0	0.0	0.0	2.0	0.0	0.0	0.0	0.055	0.22
	Genus: vadinCA02 (OTU 47)	0.0	0.0	0.0	0.0	0.0	0.35	4.4	0.17	0.0	0.3	0.33	0.14
	Kingdom: Bacteria (OTU 52)	2.0	0.41	0.0	0.5	0.43	0.26	0.0	0.0	0.0	0.0	0.22	0.0
	Kosmotoga mrcj (OTU 35)	0.6	0.64	0.3	0.45	2.2	2.7	0.0	0.0	0.0	0.0	0.77	0.0
	Order: Acidimicrobiales (OTU 116)	0.0	0.0	0.0	0.0	0.0	0.0	2.0	0.057	0.0	0.0	0.055	0.0
	Order: Actinomycetales (OTU 16)	3.9	0.47	0.0	8.5	0.27	4.7	0.0	0.0	0.0	0.0	0.22	0.0
	Order: Actinomycetales (OTU 64)	4.0	0.0	0.0	0.0	0.054	0.0	0.0	0.0	0.0	0.0	0.055	0.0
	Order: Actinomycetales (OTU 82)	0.6	0.18	0.0	1.1	0.0	0.7	0.16	0.0	0.0	0.0	0.49	0.0
	Order: Bacteroidales (OTU 34)	0.0	2.3	0.0	0.4	1.0	0.0	0.0	0.0	0.0	0.0	2.7	0.0
	Order: Bacteroidales (OTU 73)	2.6	0.0	0.0	0.2	0.054	0.18	0.0	0.0	0.0	0.0	0.11	0.0
	Order: Bacteroidales (OTU 88)	1.4	0.53	0.0	0.15	0.16	0.0	0.9	0.0	0.0	0.0	0.055	0.0
	Order: Clostridiales (OTU 189)	0.0	0.0	0.0	0.0	0.0	0.0	1.1	0.0	0.0	0.0	0.0	0.0
	Order: Clostridiales (OTU 203)	0.0	0.0	0.042	0.0	0.0	0.0	0.0	0.0	1.2	0.0	0.0	0.0
	Order: Clostridiales (OTU 25)	0.0	0.29	0.0	7.2	0.0	0.0	0.0	0.0	0.0	0.0	0.11	0.0
	Order: Clostridiales (OTU 493)	0.085	0.12	0.0	2.0	0.0	0.18	2.6	2.5	0.0	2.5	0.82	1.4
	Order: Clostridiales (OTU 75)	0.0	0.0	0.0	0.0	0.0	0.0	0.0	0.0	4.5	0.0	0.0	0.0
	Order: Clostridiales (OTU 97)	0.0	0.82	0.0	0.0	0.054	0.0	0.0	0.0	0.0	0.0	1.0	0.0
	Order: GAB-B06 (OTU 72)	0.0	0.0	0.0	0.0	2.0	0.0	0.0	0.0	0.0	0.23	0.0	0.0
	Order: GCA004 (OTU 45)	0.85	1.9	0.0	0.25	0.32	0.0	0.0	0.0	0.0	0.0	1.3	0.0
	Order: JG30-KF-CM45 (OTU 140)	0.0	0.0	0.0	0.0	0.0	0.0	1.1	0.11	0.0	0.067	0.0	0.0
	Order: LD1-PB3 (OTU 141)	0.0	0.0	0.0	0.0	0.0	0.0	1.5	0.0	0.0	0.0	0.0	0.0
	Order: Rhizobiales (OTU 121)	0.0	0.0	0.0	0.0	0.0	0.0	1.9	0.0	0.0	0.0	0.0	0.0
	Order: Rhizobiales (OTU 148)	0.0	0.0	0.0	0.0	0.0	0.0	1.8	0.0	0.0	0.0	0.0	0.0
	Order: Rhizobiales (OTU 262)	0.0	0.0	0.0	0.0	0.0	0.0	1.1	0.0	0.0	0.0	0.0	0.0
	Order: SJA-15 (OTU 1575)	22	18	0.0	1.2	10	3.0	0.0	0.0	0.0	0.0	14	0.024
	Order: SJA-15 (OTU 231)	1.4	0.59	0.0	0.2	0.32	0.26	0.0	0.0	0.0	0.0	0.49	0.0
	Order: Thermoanaerobacterales (OTU 182)	0.0	0.0	0.25	0.0	0.0	0.0	0.0	0.0	1.8	0.0	0.0	0.0
	Phylum: Chloroflexi (OTU 24)	0.94	2.5	0.0	0.8	2.2	3.8	0.0	0.0	0.0	0.0	1.6	0.0
	Unknown (OTU 130)	0.0	0.0	0.0	0.0	0.0	0.0	1.8	0.0	0.0	0.0	0.0	0.0
	Unknown (OTU 176)	0.0	0.0	0.0	0.0	0.0	0.0	1.5	0.0	0.0	0.0	0.0	0.0
	Unknown (OTU 223)	0.0	0.0	0.0	0.0	0.0	0.0	1.1	0.0	0.0	0.0	0.0	0.0
	Unknown (OTU 31)	0.17	1.0	2.9	1.1	1.8	1.8	0.0	0.0	0.0	0.0	1.0	0.0
	Unknown (OTU 36)	0.0	0.0	9.7	0.0	0.0	0.0	0.0	0.0	0.0	0.0	0.0	0.0
	Unknown (OTU 43)	0.34	0.94	0.0	1.1	0.59	1.1	0.0	0.0	0.0	0.0	0.49	0.0
	Unknown (OTU 61)	0.0	0.0	0.0	0.0	0.0	0.0	0.0	0.0	5.7	0.0	0.0	0.0
