# Supplementary material for: Effects of sludge inoculum and organic feedstock on active microbial communities and methane yield during anaerobic digestion
Source: Front Microbiol. 2015 Oct 13;6:1114. doi: 10.3389/fmicb.2015.01114 (PMC4602121; doi:10.3389/fmicb.2015.01114)
Supplement: Supplementary file 2 [file Image_1.PDF]

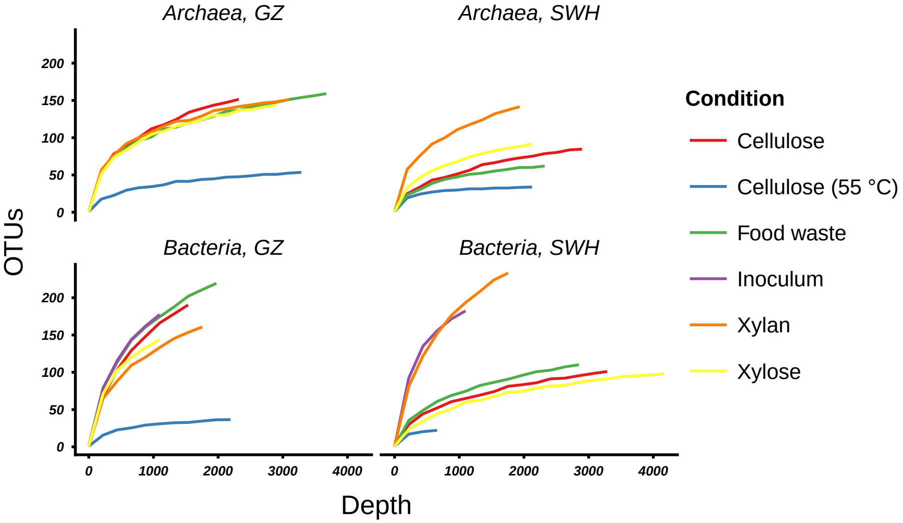

Figure S1: Rarefaction curves for each sample and each targeted domain. For each domain, twenty subsampling depths were selected between one and the depth of the deepest sequenced sample. Each sample was randomly subsampled to each depth ten times and the averaged results are displayed.
